# UNCEMENTED ARTHROPLASTY AFTER HIP METASTATIC DISEASE AND MULTIPLE MYELOMA

**DOI:** 10.1590/1413-785220162404158362

**Published:** 2016

**Authors:** André Mathias Baptista, Sergio Pinheiro de Souza Meirelles, Daniel César Seguel Rebolledo, Luiz Filipe Marques Correia, Olavo Pires de Camargo

**Affiliations:** 1. Universidade de São Paulo, Faculdade de Medicina, Hospital das Clínicas, Institute of Orthopedics and Traumatology, São Paulo, SP, Brazil.; 2. Instituto do Câncer do Estado de São Paulo (ICESP), São Paulo, SP, Brazil.; 3. Universidade de São Paulo, Faculdade de Medicina, Department of Orthopedics and Traumatology, São Paulo, SP, Brazil.

**Keywords:** Arthroplasty, replacement, hip. Neoplasm metastasis. Bone neoplasms

## Abstract

**Objective:**

To describe a case series using a combination of narrative, graphical exploratory analysis and Bayesian Network modeling*.*

**Methods:**

Case series with 34 patients undergoing uncemented and hybrid arthroplasty procedures secondary to hip pain or fracture secondary to metastatic disease or multiple myeloma*.*

**Results:**

The most common tumors included gastrointestinal, multiple myeloma and breast cancer. Most devices were total arthroplasty (n = 16, 84.2%) rather than partial and uncemented arthroplasty (n = 12, 63.2%) rather than hybrid. The average time between surgery and deambulation was 20 days, the average length of hospital stay was 13 days, and the average patient survival was 589 days. Only one infection was reported. Uncemented and hybrid arthroplasty devices did not differ regarding time to walk, as well as the length of hospital stay in this sample*.*

**Conclusion:**

Our model may be used as a prior for the addition of subsequent patient samples, personalizing, thus, its recommendations to other patient populations. *Level of Evidence IV, Case series.*

## INTRODUCTION

The increased survival of patients with bone metastatic disease or multiple myeloma has led to a subsequent increase in the incidence of imminent and pathologic fractures of the proximal femur in this population.^1^ As a consequence, prophylactic and therapeutic orthopedic procedures are now performed more frequently.^2^ Currently, cemented hip arthroplasty is considered the treatment of choice for such fractures.^3^ However, such procedures are associated with an increased rate of adverse events,^4^ ^,^ ^5^ specially given the common occurrence of comorbid conditions.^6^ In face of this high rate of complications, it would be plausible to consider the use of uncemented arthroplasty but, to our knowledge, there is no adequate evaluation of whether it would be a viable alternative.

Uncemented arthroplasty of the hip is a widely used method in patients with osteoporosis in the hip region, after hip fracture complications, as well as in association with osteoarthritis.^7^ Despite its higher cost, compared to traditional cemented arthroplasty, this procedure has been associated with lower operating time, more efficient medullary canal preparation and lower cardiopulmonary complication rates than cement arthroplasty.^8^ These features would be desirable among patients with metastatic disease, not only since recent series have demonstrated an overall survival improvement,^9^ but also regarding its safer profile.

In face of the potential advantages in this patient population, our study had two aims. First, to report a case series of uncemented arthroplasty cases for hip pain and fracture after metastatic disease and multiple myeloma, performed at *Instituto do Cancer do Estado de São Paulo* (ICESP) between 2010 and 2014. Second, to report the development of a Bayesian Network model to compare uncemented vs hybrid hip arthroplasty devices regarding time until the first walk and length of hospital stay. We hypothesized that uncemented devices would outperform hybrid devices.

## METHODS

Our study was approved by the Institutional Review Board (approval number CAAE 37873014.1.0000.0065), with participants providing informed consent prior to the initiation of our protocol. We also followed the recommended case series reporting guidelines.^10^

The patients in our study underwent a total or partial hip arthroplasty with uncemented stems, or hybrid procedures with a cemented cup. All procedures were performed and followed up at *Instituto do Câncer do Estado de São Paulo* (ICESP). Both the Orthopedics and Clinical Oncology staff followed each patient in an integrated clinical service.

All surgical procedures were indicated for (a) pathological fractures or (b) prophylactic treatment for metastatic lesions or multiple myeloma in the periacetabular or femoral regions. Information regarding gender, age, tumor diagnosis, medication history, and radiotherapy treatment were obtained through electronic health records.

Our final sample was composed by 18 women and 16 men with mean age of 67.1 years old (range 42-88 years old). Primary tumors showed the following distribution: nine breast cancers, one lung cancer, eight prostate cancers, seven gastrointestinal cancers, five multiple myeloma and four of other types of tumor. Of these patients, only two received preoperative radiotherapy, indicated for pain relief, and two received post-operative radiotherapy. A total of 14 patients undergoing total hip arthroplasty were admitted through the emergency room with hip fractures. All remaining patients presented with hip pain or significant disability, with an accompanying radiograph and magnetic resonance imaging (MRI).

All patients were followed with radiographs and magnetic resonance of the corresponding hip to evaluate local progression, using a 3 to 6-month interval depending on patient compliance. Bone scans were used to evaluate new lesions. Based on imaging criteria, physical exam and pain rating scores, we then indicated osteosynthesis or hip arthroplasty on a case by case basis.

All the patients included in this study underwent total or partial hip arthroplasty with a non-cemented femoral stem. There were 17 uncemented total arthroplasties, with nine of them being indicated after a fracture. In addition, nine patients received hybrid arthroplasties (cemented acetabulum and uncemented femoral stem), while eight patients received partial arthroplasties.

All procedures were performed through a lateral approach, with a 3.2mm postoperative drain that remained until the volume in the past 24 hours was below 50ml. In all procedures, a cemented arthroplasty was available in the operating room, in case of a change in the surgical plan.

Preoperative heparin (40mg subcutaneous injection) once a day was restricted to patients with a pathologic fracture that were, therefore, unable to walk before surgery. All patients received an intraoperative elastic band compression on their lower extremities combined with postoperative enoxaparin (40 mg subcutaneous) once a day for three weeks after the surgical procedure.

Outcomes quantitatively evaluated in this patient sample included length of stay (time between admission and discharge), time until death (time between the first surgery and death, with death being identified by family members), time between diagnosis and surgery, time until walking (time between surgery and first walk), postoperative infection, surgical debridement and antibiotic treatment.

### Causal and Decision Support Modeling

We initiated the analysis through a descriptive graphical analysis, with numeric variables evaluated in relation to their statistical distribution and variance. Categorical variables were evaluated for their percentages. Variables with near zero categorical variance either had their low-frequency category merged with other categories or the variable itself was not taken into consideration for further modeling. Causal modeling was conducted using a Bayesian Network. Given the small sample available for this study, we did not infer the structure from the data, but instead used a standardize protocol to extract the structure from clinical experts. This structure was, then, followed by parameter estimation for each node connection. Expert elicitation was approached by first having the lead author (SM) generate a causal graph based on existing variables. This graph was later brought to discussion with co-authors using a variant of the Delphi method. Although the final graph structure of choice was the one elicited from experts, we did attempt to infer the structure directly from the data using the following algorithms: grow-shrink, incremental association and its variants, hill-climbing, tabu search and restricted maximization, hill-climbing, tabu search and restricted maximization algorithms. For the score computation we used the following algorithms: Gaussian log-likelihood, Akaike Information Criterion, Bayesian Information Criterion, Bayesian Dirichlet equivalent and K2. An initial attempt to estimate structure using a hybrid model containing Gaussian and discrete variables resulted in poor fit. This was, therefore, followed by a second model with discrete variables. All analyses were conducted using the blearn package and the R statistical language. Additional packages included the ggplot2, tabplot, knitr, moonBook and survival.

### Statistics analysis

Our sample (Table 1) was composed by a slightly greater number of males, with a mean age around 67 years. Diagnoses were highly heterogeneous, with the most prevalent tumor being breast cancer. The majority of patients underwent total uncemented arthroplasty procedures and most did not undergo radiotherapy for pain control. Only one patient in this series evolved with infection, which was followed by debridement. Given the sparsity of our data, we represented it through a single plot, so that information on individual patients could be fully visualized. (Figure 1A and Figure 1B)

**Table 1:** Descriptive statistics for study sample.

	Total (N=34)	p
Age	67.1 Â ± 9.8 (42 - 88)	0.466
Female	18 (52.9%)	0.809
Arthroplasty		0.488
- Partial	8 (23.5%)	
- Total	26 (76.5%)	
Right Side	15 (53.6%)	0.030
Cementation		0.304
- Hybrid	9 (26.5%)	
- Non-cemented	25 (73.5%)	
Fracture	15 (44.1%)	0.063
Tumor Type		0.903
- Bladder	1 (2.9%)	
- Breast	9 (26.5%)	
- Gastrointestinal	7 (20.6%)	
- Glioblastoma	1 (2.9%)	
- Lung	1 (2.9%)	
- Lymphoma	1 (2.9%)	
- Melanoma	1 (2.9%)	
- Multiple Myeloma	5 (14.7%)	
- Prostate	8 (23.5%)	
Length Of Stay	14.3 Â ± 24.2 (42 - 114)	0.954
Death	15 (44.1%)	0.809
Time Until Death	425.8 Â ± 388.5 (6 - 1419)	0.019
Radiotherapy		1.000
- No	30 (88.2%)	
- Post	2 (5.9%)	
- Pre	2 (5.9%)	
Walking Time	12.6 ± 19.1 (0 - 82)	0.030
Infection	1 (2.9%)	0.560
Surgical Debridement	1 (2.9%)	0.560
Antibiotic Treatment (Ciprofloxacin/Pseudomonas)	1 (2.9%)	0.560

**Figure 1A:**
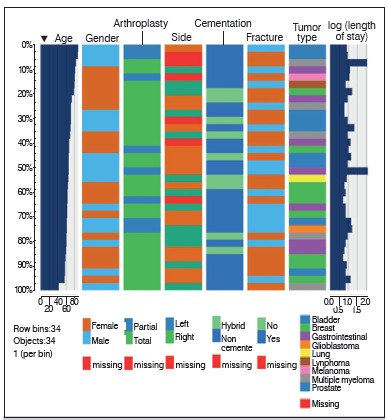
Exploratory graphical analysis of the study sample.

**Figure 1B:**
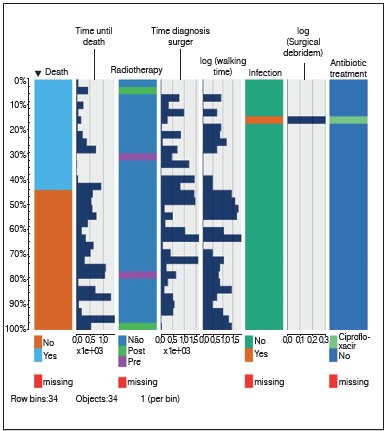
Exploratory graphical analysis of the study sample.

**Figure 2:**
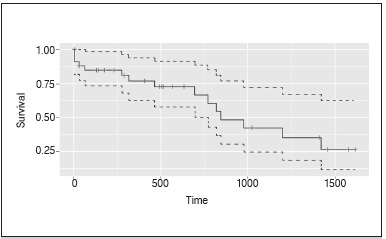
Time between diagnosis and surgery.

**Figure 3:**
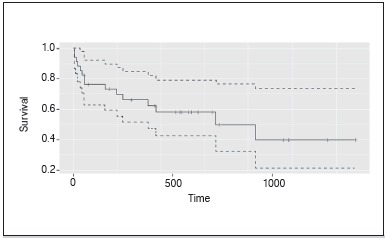
Patient survival.

Surgical procedures largely occurred during the first two years after diagnosis, and only 4 patients were operated on the first week after diagnosis. Surgeries were performed up to 4.4 years after diagnosis. (Figure 2) Fifteen patients died during the course of the study, their mortality was distributed as displayed in Figure 3.

**Figure 4:**
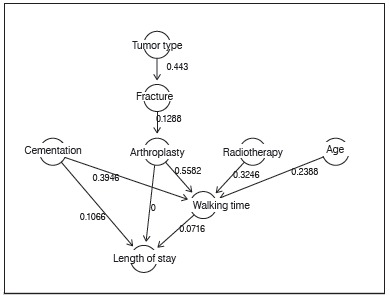
Causal model structure and parameters.

Figure 4 represents our causal model where its structure was based on expert opinion and where parameters connecting different variables were inferred directly from the data. The causal model with probability parameters inferred from our data is displayed in Figure 4. Of all associations, the effect of the tumor type on the probability of fracture accounted for the largest conditional probability.

**Figure 5:**
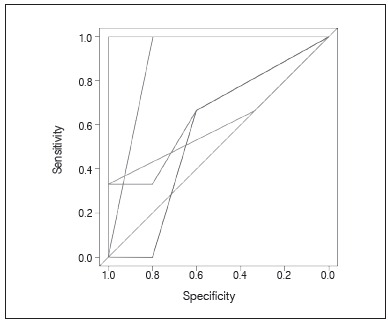
ROC for the Bayesian Network model.

Predictive performance for our model achieved an area under the curve of 0.74, which can be considered as fair. (Figure 5) Of central importance to our study is the fact that uncemented and hybrid arthroplasty devices did not differ in relation to time to walk, as well as length of stay in this sample. (Table 2)

**Table 2:** Experiments comparing uncemented versus hybrid arthroplasty in relation to probability of walking and length of stay.

	Total	Hybrid	Uncemented
(11.8.114)	0.2454978	0.2787378	0.2330328
(6.8)	0.2530978	0.4870868	0.1653519
(8.11.8)	0.1196554	0.0568182	0.1432194
(4.6)	0.3817490	0.1773573	0.4583959
(0.0.5)	0.2283314	0.2787378	0.2094290
(0.5.5.5)	0.1906461	0.2116829	0.1827573
(5.5.13)	0.2279387	0.2116829	0.2340346
(13.82)	0.3530838	0.2978963	0.3737791

Impact of Cementation on Time Until Walking.

## DISCUSSION

To our knowledge, this is the first comprehensive case report, graphical exploratory analysis and causal modeling related to the use of uncemented arthroplasty for metastatic disease and multiple myeloma of the hip. We have described our case series in relation to its clinical outcomes, but we also described a Bayesian Network model connecting several clinical factors to walking time and length of stay. Specifically, an experiment conducted within this model demonstrated that uncemented and hybrid arthroplasty devices did not differ in relation to time to walk, as well as the length of stay in this sample.

To date, cemented components are the traditional surgical technique when a hip arthroplasty is indicated for pain or fracture after metastatic disease or multiple myeloma in the hip area.^3^ ^,^ ^11^ ^-^ ^13^ Previous studies have described the use of composite allograft with uncemented arthroplasty in the treatment of primary bone tumors,^14^ arguing that the cardiovascular risks associated with the use of cement could be avoided. This is also a strong argument in relation to patients with metastatic disease and multiple myeloma, as their overall health status is frequently compromised. An additional argument includes the increased complexity of revision procedures if they might ever be needed. While in the past one could simply dismiss this point as survival tended to be poor, recent improvements in therapeutic management^9^ ^,^ ^15^ now warrant reconstruction procedures where revision should be considered. Besides these former arguments, uncemented procedures use an interference or press fit mechanism, allowing for bone ingrowth and revascularization once weight bearing is initiated.^16^ ^-^ ^18^

While our case series made use of a reporting guideline^10^ to ensure that patient information can be more reliably interpreted by research and clinical peers, the narrative nature of case series does not allow for substantive evidence accumulation.^19^ To decrease this limitation, we used a Bayesian Network model that combines expert knowledge in form of its structure, while the relationship among different clinical factors is directly inferred from data.^20^ Bayesian Networks allow for a number of advantages while being used in the context of case series, namely, the ability to develop formal models with small sample sizes, good predictive performance, the possibility of adding data from other case series, as well as the literature to generate cumulative evidence and, finally, the possibility of creating decision support tools such as Web applications so that model results can be accessed at the bedside.^21^

Despite its novelty and significance, our study does have some limitations. First, our sample is acknowledgedly small. Despite its size, our models using Bayesian Networks can be expanded through the addition of other case series, thus, allowing for cumulative evidence rather than remaining as an isolated narrative. Second, our model might not have accounted for all possible confounding factors affecting the association between uncemented arthroplasty and time to walk as well as length of stay, ultimately not substituting a randomized experiment.^20^ While this is certainly a limitation, our model allowed us to establish an experiment comparing uncemented vs. hybrid arthroplasty procedures while having this experiment taking into account the values of all parent nodes, which helps decreasing confounding factors.^22^ Last, given our limitations in terms of sample size, all of our model variables had to be categorized in order to have an acceptable predictive performance.^20^ Although maintaining some of the clinical variables in its original numeric values would have provided a higher degree of information, future models with larger patient samples might use our estimates as Bayesian priors.

## CONCLUSION

In sum, based on this case series with an accompanying Bayesian Network model, we have found that uncemented arthroplasty devices increase the conditional probability of both earlier time to walk as well as decreased length of stay among patients with metastatic disease or multiple myeloma in the hip area. Given the small sample in our study, we encourage other researchers to update our model results through their local data, thus, not only increasing the body of evidence-based knowledge for this procedure, but by also localizing its results to their population of interest.
